# Genome-Wide Association Mapping of Grain Protein Content and Haplotype Analysis in Cold-Region Japonica Rice

**DOI:** 10.3390/plants15213355

**Published:** 2026-10-29

**Authors:** Yingying Liu, Liangzi Cao, Guohua Ding, Jinsong Zhou, Lei Lei, Baojia Han, Dawei Gao, Li Wen, Shichen Sun

**Affiliations:** 1 Institute of Crop Cultivation and Tillage, Heilongjiang Academy of Agricultural Sciences, Harbin 150086, China; 2 Heilongjiang Rice Quality Improvement and Genetic Breeding Engineering Research Center, Harbin 150086, China; 3 School of Agriculture, Northeast Agricultural University, Harbin 150030, China; 4 Biotechnology Research Institute, Heilongjiang Academy of Agricultural Sciences, Harbin 150023, China; 5 Inner Mongolia Key Laboratory of Rice Breeding Innovation in Northern Cold Regions, Hinggan League Academy of Agricultural and Animal Husbandry Sciences, Ulanhot 137400, China

**Keywords:** cold-region japonica rice, grain protein content, genome-wide association study, haplotype, eating quality

## Abstract

Grain protein content (GPC) is an important determinant of rice nutritional and eating quality. In this study, 624 cold-region japonica rice accessions were evaluated for GPC and taste value at Harbin Minzhu, Ulanhot, and a saline–alkali site in the Songnen Plain, and 495 accessions with complete phenotypic and genotypic data were used for genome-wide association analysis. GPC showed significant positive correlations across sites and an entry-mean broad-sense heritability of 0.663. Using 1,527,603 high-quality SNPs, GLM-Q identified 56 significant GPC-associated SNPs, mainly on chromosomes 11 and 7, with site-specific patterns. Annotation of GLM-Q significant SNPs yielded 28 non-redundant positional candidate genes. In GEMMA, chromosome 11 signals did not remain genome-wide significant, whereas chromosome 7 associations remained significant. Haplotypes of *Os11g0239500*, *Os11g0239900*, *Os07g0264100*, and *Os03g0244200* differed in GPC and taste value. Joint haplotype analysis of *Os11g0239500* and *Os07g0264100* classified 319 accessions into four types. Type IV had the lowest mean GPC (7.84%) and the highest mean taste value (81.14), whereas Type I had a mean GPC of 8.78% and a mean taste value of 77.45. The identified candidate genes and haplotypes may be useful for screening cold-region japonica rice with low GPC and high eating quality.

## 1. Introduction

Improving rice quality requires the simultaneous consideration of nutritional value and eating quality. Grain protein content (GPC) is an important component of rice nutritional quality, but excessively high GPC is generally associated with increased hardness and reduced palatability of cooked rice. Previous studies have shown that rice protein content and composition are closely related to eating quality [1,2,3]. Agronomic management, including nitrogen and potassium fertilization, can further alter protein accumulation and eating quality [1,4,5]. More importantly, recent genetic studies have demonstrated that natural variation can simultaneously affect GPC and eating quality. For example, a favorable haplotype of *Ghd7.1* was reported to reduce GPC while improving eating quality [6]. Related work has shown that *Ghd7* can also coordinate carbon and nitrogen metabolism to improve grain quality and yield [7]. These findings indicate that coordinated improvement of GPC and eating quality may be achieved through the selection of favorable genetic variation rather than through agronomic regulation alone.

GPC is a complex quantitative trait controlled by multiple physiological and molecular processes, including nitrogen uptake and assimilation, amino acid transport, and storage protein biosynthesis and accumulation [8]. Several genes involved in these processes have been characterized in rice. *OsAAP6* encodes an amino acid transporter that contributes to amino acid allocation to developing grains and influences grain protein accumulation [9,10]. Loss of *OsAAP11* function has also been shown to reduce GPC and improve certain aspects of eating quality [11]. In addition, natural variation in the promoter region of *OsGluA2*, which encodes a glutelin A2 precursor, is associated with altered gene expression and variation in GPC [12]. Recent work further showed that transcriptional regulation of *OsGluA2* by the *OsGATA7*-*SMOS1* module contributes to rice taste quality formation [13]. Beyond these cloned genes, numerous quantitative trait loci (QTLs) and genomic regions associated with GPC have been identified in different rice populations [14,15]. However, the effects and detectability of these loci often vary across genetic backgrounds and growing environments, highlighting the importance of evaluating GPC associated variation within specific breeding populations.

Cold-region japonica rice has undergone long term selection for early maturity, cold adaptation, yield stability, and eating quality, resulting in a distinct genetic background compared with rice populations from warmer production regions. Considerable variation remains in eating and cooking quality traits and in the allelic composition of major quality related genes within northeastern japonica germplasm [16,17,18]. Genome-wide association studies (GWASs), particularly when combined with linkage disequilibrium (LD) and haplotype analyses, have been widely used to dissect the genetic basis of grain appearance, milling quality, eating and cooking quality, and other complex traits in rice [19,20,21,22,23,24]. Nevertheless, the genetic basis of GPC variation in cold-region japonica rice remains insufficiently characterized. In particular, it is unclear whether major GPC-associated regions show consistent or site-dependent effects across contrasting production environments, and whether natural variation within these regions is associated with simultaneous changes in GPC and eating quality.

**Figure 1:**
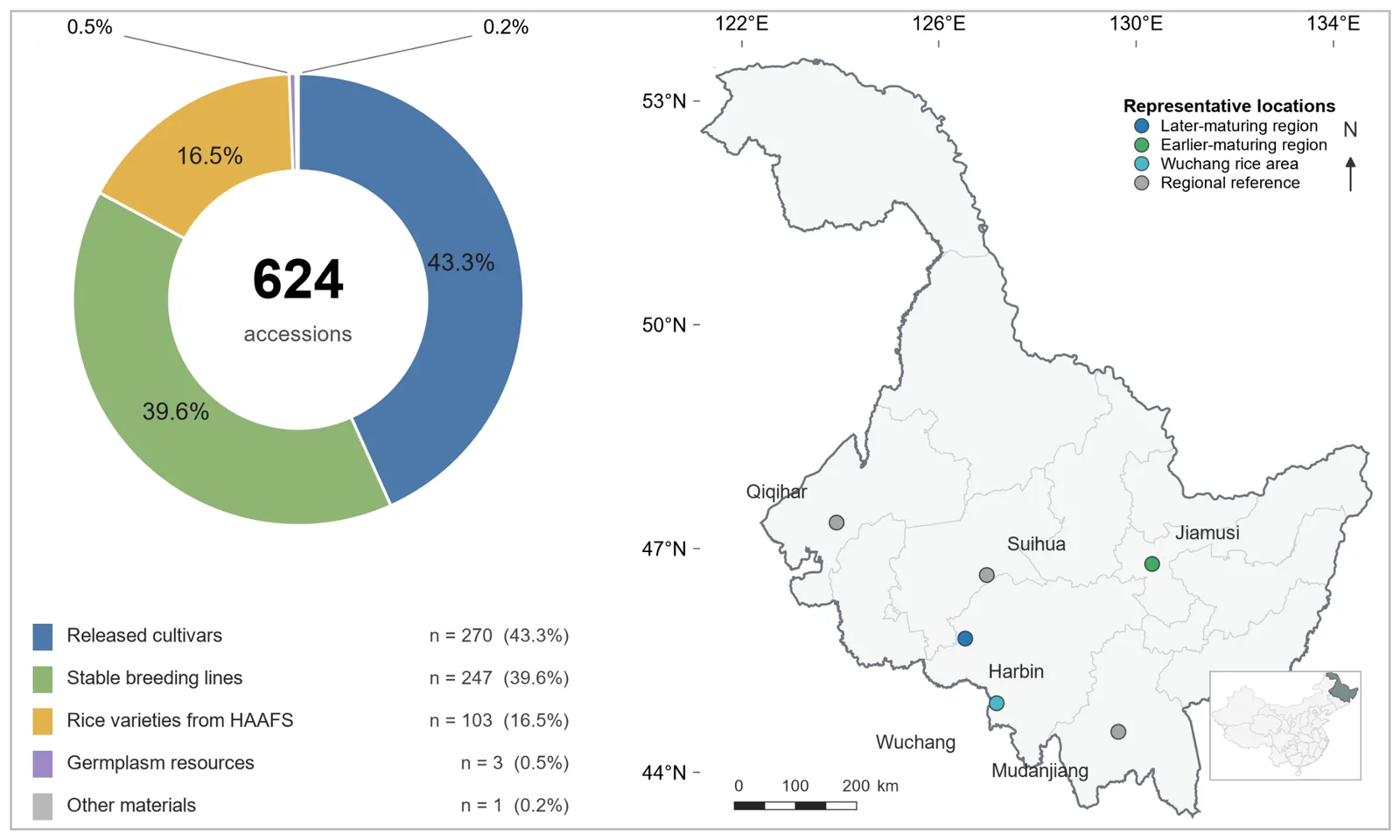
Composition of the 624 cold-region japonica rice accessions and representative rice-growing locations in Heilongjiang Province.

In the present study, 624 cold-region japonica rice accessions (Figure 1) were evaluated for GPC and eating quality at three experimental sites: Harbin Minzhu, Ulanhot, and a saline–alkali site in the Songnen Plain. Among them, 495 accessions with complete phenotypic and genotypic data were used for GWASs. Candidate genes were identified from the annotation results of significant GWAS SNPs and further evaluated through haplotype analysis, together with previously reported quality- and GPC-related genes. By comparing GPC associated signals across experimental sites and examining the phenotypic effects of haplotypes, this study aimed to identify favorable genetic variation associated with reduced GPC and improved eating quality and to provide useful haplotype resources for quality oriented breeding of cold-region japonica rice.

## 2. Results

### 2.1. Phenotypic Variation in GPC

**Figure 2:**
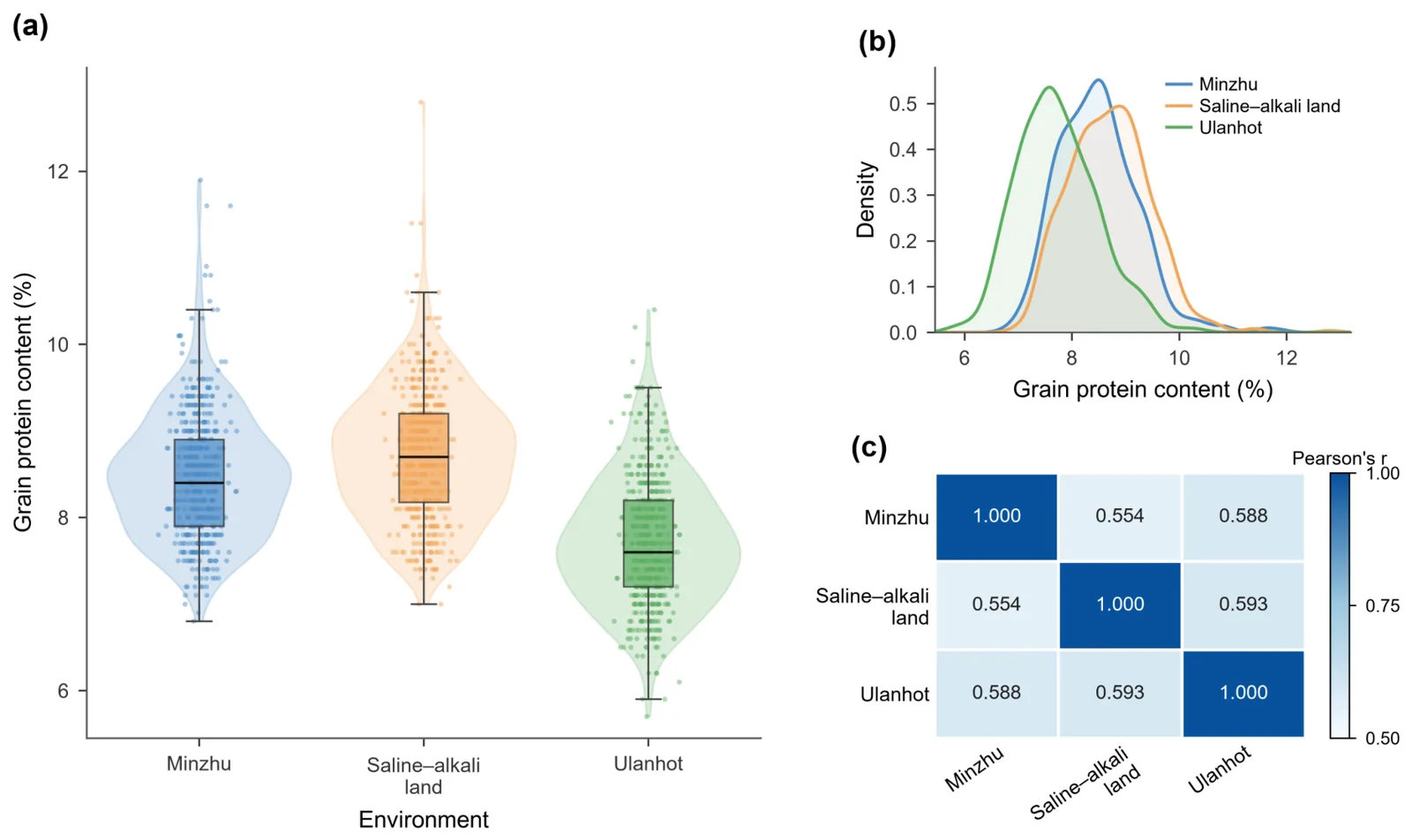
Phenotypic variation in grain protein content across three experimental sites. (**a**) Distribution of grain protein content (GPC). (**b**) Kernel density distributions of GPC. (**c**) Pearson correlation coefficients for GPC among the three experimental sites. In (**a**), dots represent individual observations, colored violins show distributions, and box plots summarize GPC. In (**b**), blue, orange, and green curves represent GPC densities for Minzhu, the saline–alkali site, and Ulanhot, respectively. In (**c**), shading indicates Pearson correlation coefficients.

Of the 624 cold-region japonica rice accessions, 495 had complete GPC data across all three experimental sites and were used for GWASs. GPC exhibited continuous variation at Harbin Minzhu, Ulanhot, and the saline–alkali site (Figure 2). The mean GPC values were 8.48%, 7.73%, and 8.70%, respectively, with corresponding standard deviations of 0.73, 0.75, and 0.76. GPC ranged from 6.80% to 11.90% at Harbin Minzhu, from 5.70% to 10.40% at Ulanhot, and from 7.00% to 12.80% at the saline–alkali site. The coefficients of variation ranged from 8.62% to 9.70%, indicating substantial phenotypic variation within the population (Table 1).

Significant positive correlations in GPC were observed among the three experimental sites (Figure 2). The Pearson correlation coefficients were 0.588 between Harbin Minzhu and Ulanhot, 0.554 between Harbin Minzhu and the saline–alkali site, and 0.593 between Ulanhot and the saline–alkali site. The entry-mean broad-sense heritability of GPC across the three experimental sites was estimated to be 0.663 using the REML-based mixed model.

### 2.2. Population Structure and Linkage Disequilibrium

After genotype quality control, 1,527,603 high-quality SNPs were retained for the 495 accessions included in the GWASs. ADMIXTURE cross-validation analysis showed a progressive decrease in CV error from K = 1 to K = 15, with the lowest value observed at K = 15. Therefore, K = 15 was selected to characterize population structure. Linkage disequilibrium (LD) decreased progressively with increasing physical distance between markers, and the LD decay distance was approximately 300 kb, defined as the distance at which the mean *r*^2^ declined to half of its initial value.

**Figure 3:**
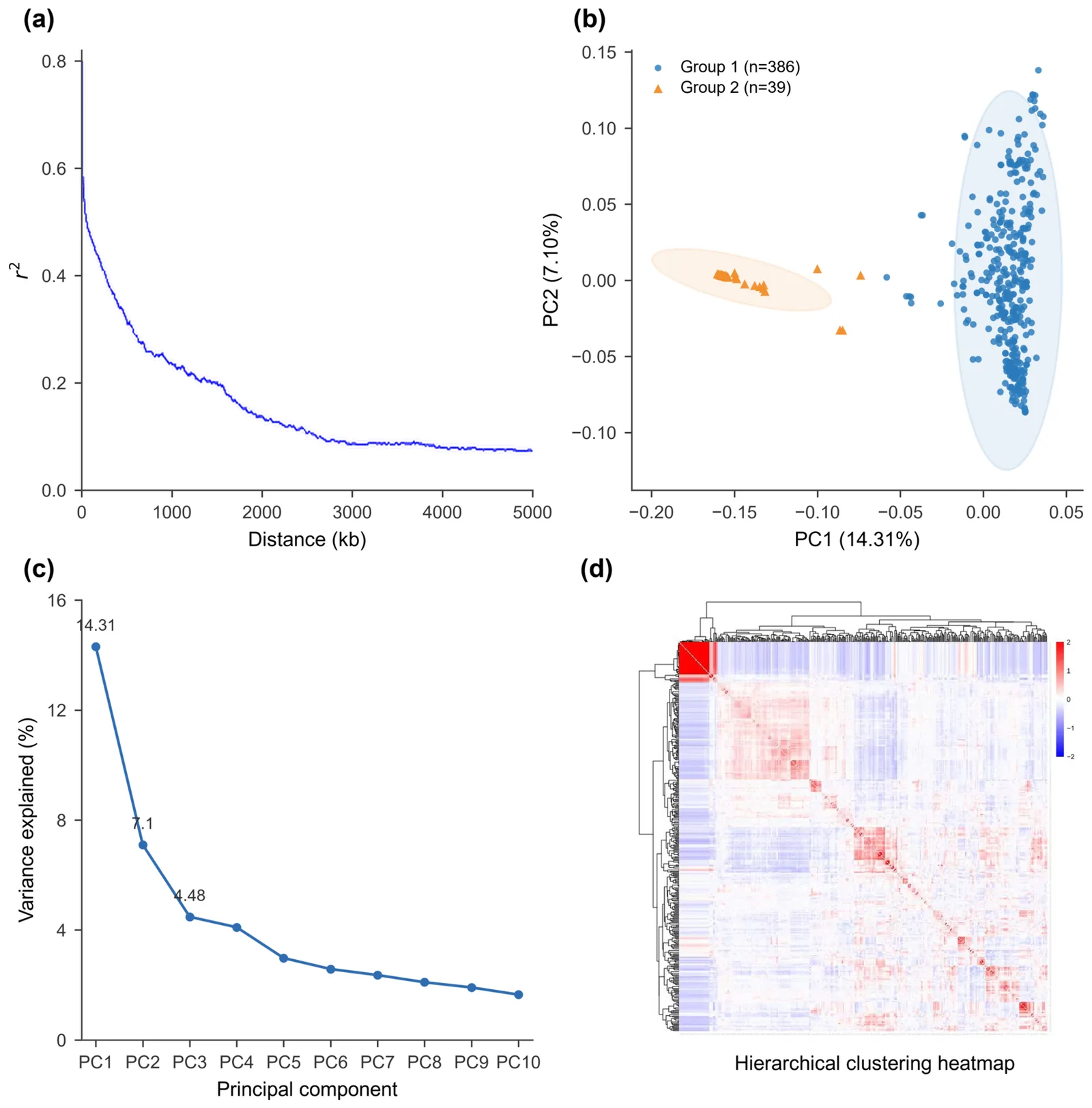
Linkage disequilibrium, population structure, and genetic relationships of the GWAS panel. (**a**) Linkage disequilibrium decay. (**b**) Principal component analysis (PCA). (**c**) Variance explained by the first ten principal components. (**d**) Kinship matrix displayed as a hierarchical clustering heatmap. In (**b**), blue and orange points and shaded ellipses represent Group 1 (n = 386) and Group 2 (n = 39), respectively. In (**d**), the color scale represents the values in the kinship matrix.

For PCA, 70 of the 495 accessions included in the GWASs were excluded because their individual genotype missingness exceeded 10%, leaving 425 accessions for PCA. PC1, PC2, and PC3 explained 14.31%, 7.10%, and 4.48% of the total genetic variation, respectively, accounting for 25.89% in combination (Figure 3b,c). The PCA based on PC1 and PC2 revealed an overall continuous distribution of the accessions, although a subset was separated along PC1, suggesting the presence of two major genetic groups (Figure 3b). The first ten principal components collectively explained 43.58% of the total genetic variation (Figure 3c). Kinship analysis further showed generally low pairwise relatedness among most of the 495 accessions, whereas relatively high genetic similarity was restricted to a limited subset of the population (Figure 3d).

### 2.3. GWAS of Grain Protein Content

**Figure 4:**
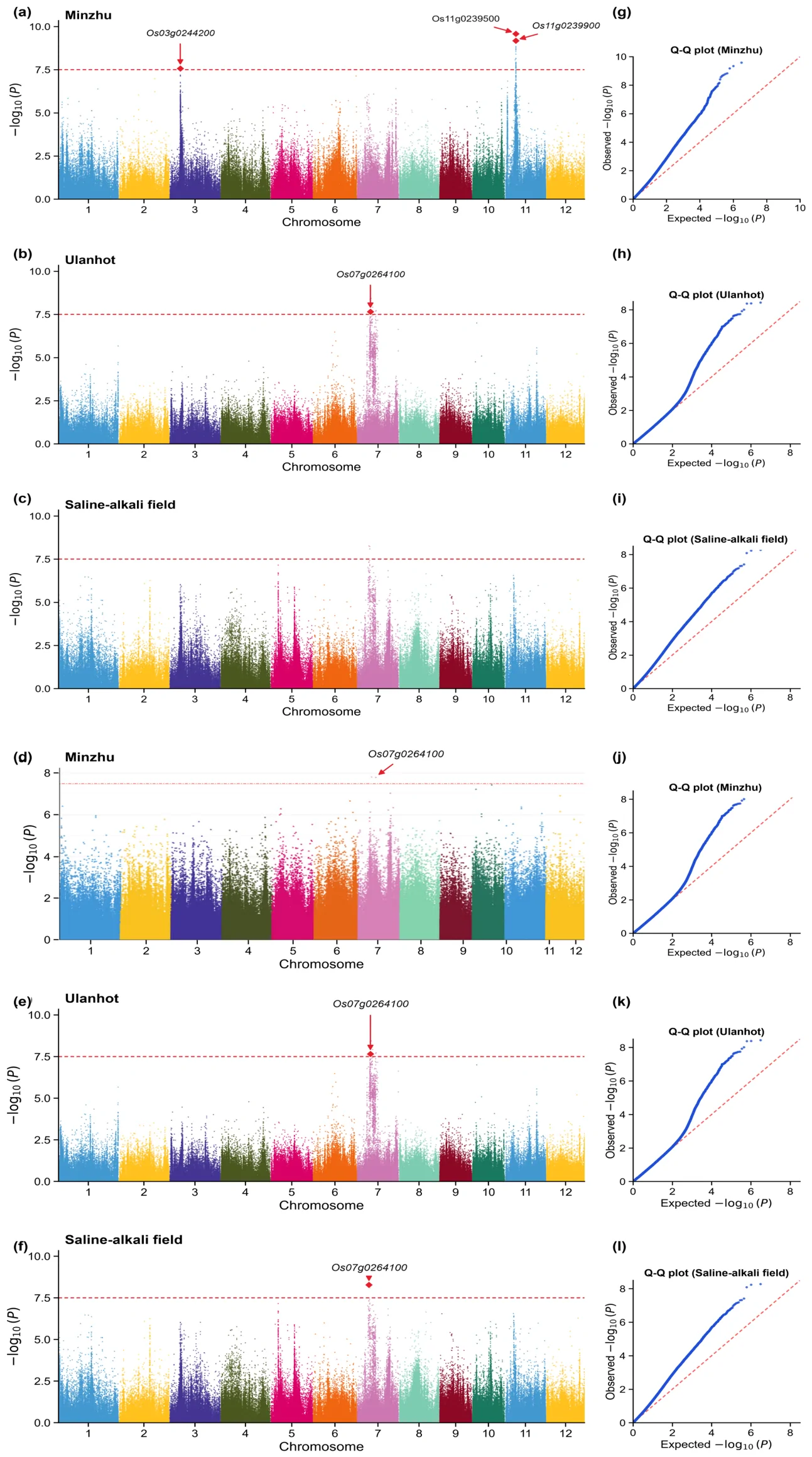
Genome-wide association analysis of grain protein content across three environments using GLM and GEMMA models. (**a**–**c**) Manhattan plots from the GLM analyses of Minzhu, Ulanhot and the saline–alkali field, respectively; (**g**–**i**) the corresponding Q–Q plots. (**d**–**f**) Manhattan plots from the GEMMA analyses of the same environments, respectively; (**j**–**l**) the corresponding Q–Q plots. Each point represents a SNP, and the y-axis shows $-\log_{10}\left(P\right)$. The red dashed line indicates the significance threshold of $-\log_{10}\left(P\right)>7.5$ ($p<3.16\times 10^{-8}$). Arrows mark selected association signals, and text labels identify candidate genes. In the Manhattan plots, dot colors distinguish chromosomes and red diamonds highlight selected SNPs. In the Q–Q plots, blue dots show observed values and red dashed diagonals indicate null expectations.

Genome-wide association analyses were conducted separately for the three experimental sites. At a genome-wide significance threshold of −log_10_(*p*) > 7.5, a total of 56 SNPs were significantly associated with GPC (Figure 4). Among these, 31, 19, and 6 significant SNPs were detected at Harbin Minzhu, Ulanhot, and the saline–alkali site, respectively. The significant SNPs were distributed across chromosomes 3, 6, 7, 10, and 11, with most signals concentrated on chromosomes 11 and 7. Specifically, 30 significant SNPs were located on chromosome 11 and 23 on chromosome 7, whereas chromosomes 3, 6, and 10 each contained a single significant SNP.

At Harbin Minzhu, significant SNPs on chromosome 11 were concentrated at 7.43–7.50 Mb, with an additional significant SNP at 6,915,934 bp (Figure 4). In contrast, association signals on chromosome 7 were detected predominantly at Ulanhot and the saline–alkali site. At Ulanhot, a significant intragenic variant at 9,353,384 bp was detected in *Os07g0264100*. The Q-Q plots of the GLM-Q analysis showed deviations from the expected distribution, particularly at Harbin Minzhu and Ulanhot (Figure 4). The genomic inflation factors (λGC) were 1.442, 1.331, and 1.419 at Harbin Minzhu, Ulanhot, and the saline–alkali site, respectively. In comparison, the corresponding λGC values obtained using GEMMA were 0.964, 0.955, and 0.940, respectively, indicating reduced genomic inflation and improved calibration of the association test statistics. The additional GEMMA analysis revealed differences in the association patterns compared with GLM-Q. Several signals identified by GLM-Q, particularly those on chromosome 11, no longer reached genome-wide significance. However, significant associations on chromosome 7 were detected by GEMMA at all three experimental sites (Figure 4). These results indicate that chromosome 7 retained significant associations under the mixed-model analysis, whereas the prominent chromosome 11 signals were more sensitive to model selection.

**Figure 5:**
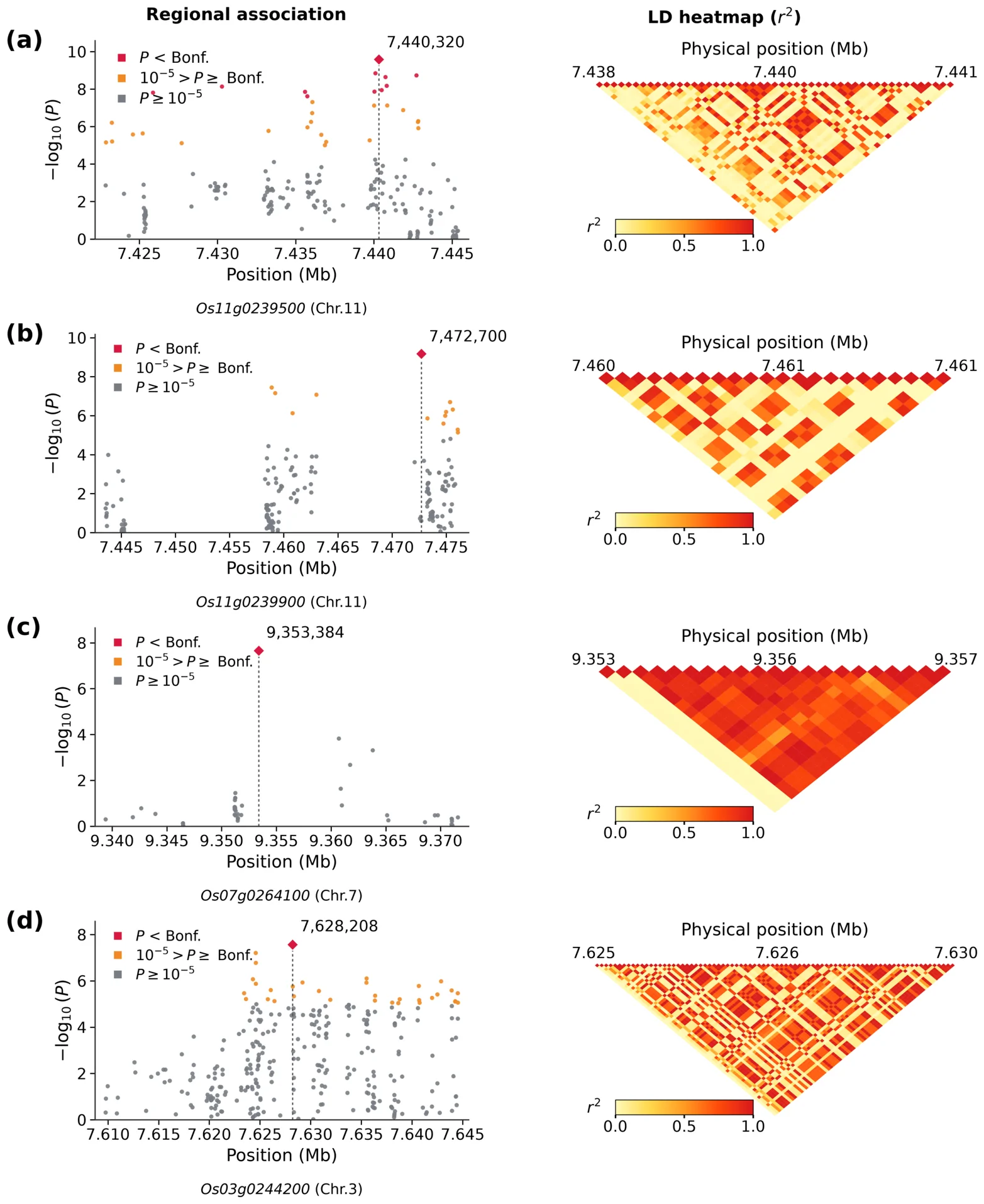
Regional association and linkage disequilibrium analyses of four candidate loci associated with grain protein content. (**a**–**d**) *Os11g0239500, Os11g0239900, Os07g0264100*, and *Os03g0244200*, respectively. **Left** panels show regional association signals, and **right** panels show local linkage disequilibrium patterns. In the regional association plots, gray, orange, and red points indicate the significance categories shown in the legends. Red diamonds highlight selected variants, and vertical dashed lines mark their genomic positions. In the LD heatmaps, the yellow-to-red color scale indicates increasing linkage disequilibrium ($r^{2}$).

**Figure 6:**
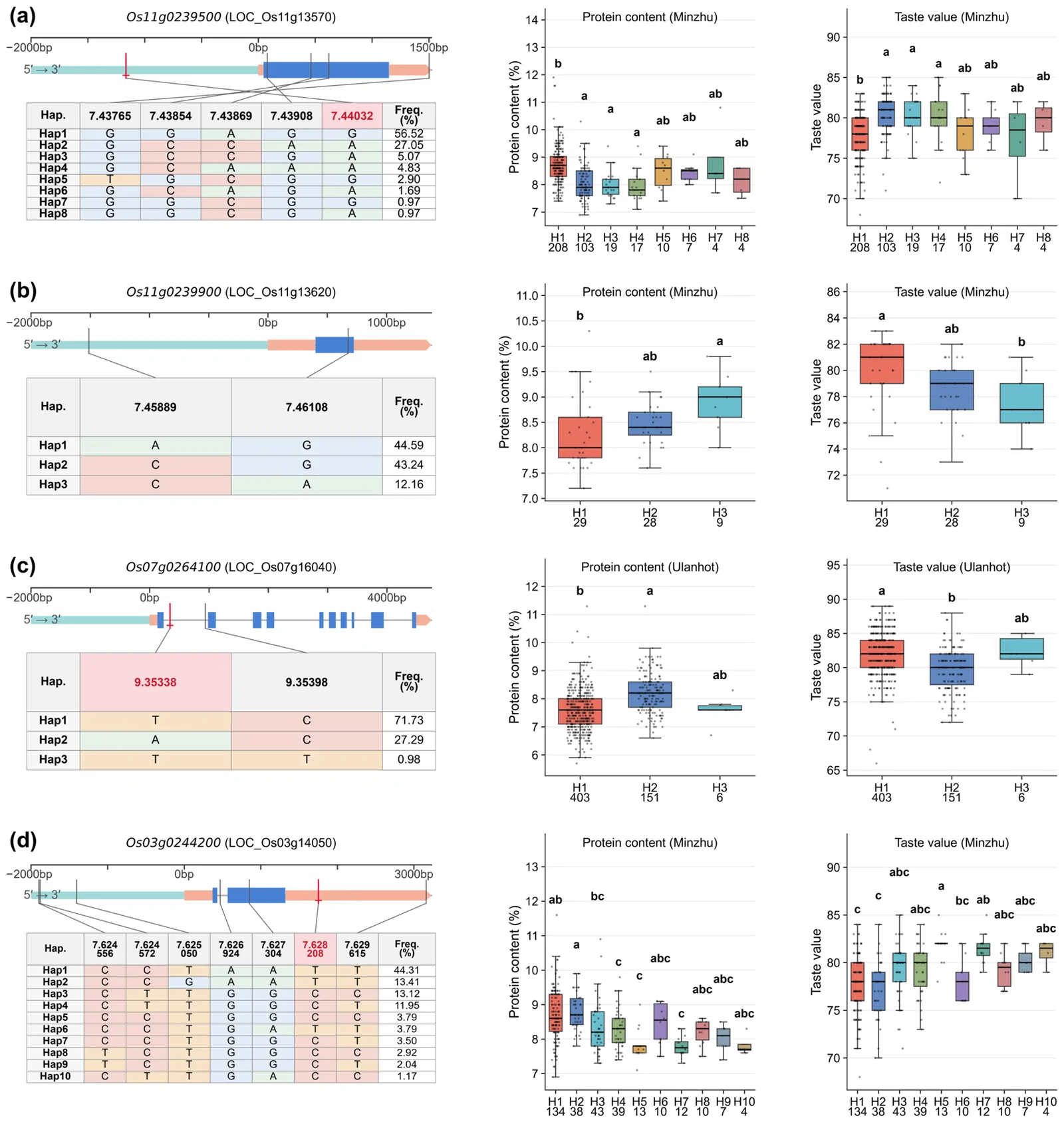
Haplotype variation and phenotypic effects of four candidate genes for GPC. (**a**–**d**) *Os11g0239500*, *Os11g0239900*, *Os07g0264100*, and *Os03g0244200*, respectively. Gene structures, haplotypes, and corresponding differences in GPC and taste values are shown. Different lowercase letters indicate significant differences at *p* < 0.05. Colored cells in the haplotype tables distinguish allele patterns. Box colors distinguish haplotype groups within each panel, and dots represent individual observations.

Based on the annotation of significant GWAS SNPs, 28 non-redundant positional candidate genes were identified (Table 2). Four representative genes were selected based on association strength, genomic distribution, and variant annotation: *Os11g0239500* and *Os11g0239900* from the major chromosome 11 association region, *Os07g0264100* containing a significant intronic SNP on chromosome 7, and *Os03g0244200* containing a significant intronic SNP on chromosome 3. These genes were further examined by regional association and local LD analyses (Figure 5), followed by haplotype analysis (Figure 6).

### 2.4. Haplotype Analysis of Candidate Genes

The phenotypic effects of haplotypes at the four candidate genes varied among the three experimental sites. For the chromosome 11 candidate genes *Os11g0239500* and *Os11g0239900*, significant haplotype differences were observed primarily at Harbin Minzhu (Figure 6). Eight haplotypes were identified for *Os11g0239500*, with significant differences in both GPC and taste value among haplotypes (Figure 6a). Hap2, Hap3, and Hap4 showed relatively low GPC values of 8.05%, 7.98%, and 7.98%, respectively, together with relatively high taste values of 80.36, 80.42, and 80.12. At Ulanhot, no clear haplotype differences were observed. At the saline–alkali site, however, Hap1 showed significantly higher GPC than Hap2, Hap3, and Hap4, and some haplotypes also differed in taste value (Figure S6a).

In a separate PC- and kinship-adjusted sensitivity analysis using stricter genotype-validation and haplotype-frequency criteria, only Hap1 and Hap2 of *Os11g0239500* were eligible. The overall haplotype associations at Harbin Minzhu remained significant for GPC (BH-adjusted *q* = 0.027) and instrument-derived taste value (*q* = 0.025), with estimated Hap2-Hap1 differences of −0.466 percentage points and 1.961 points, respectively. The corresponding adjusted overall associations were not significant at Ulanhot or the saline–alkali site (Table S2).

Three haplotypes were identified for *Os11g0239900*, with significant phenotypic differences detected at Harbin Minzhu (Figure 6b). Hap1 exhibited the lowest GPC (8.27%) and the highest taste value (79.83), whereas Hap3 showed the highest GPC (8.91%) and the lowest taste value (77.33). For *Os07g0264100*, three haplotypes were identified, and their phenotypic differences were most evident at Ulanhot (Figure 6c). Hap1, Hap2, and Hap3 had GPC values of 7.43%, 8.04%, and 7.60%, respectively, with corresponding taste values of 80.26, 78.10, and 82.33. Hap1 showed significantly lower GPC and a higher taste value than Hap2, whereas Hap3 showed the same numerical trend but had an intermediate multiple comparison grouping. This pattern was consistent with the chromosome 7 association signals detected at Ulanhot. Ten haplotypes were retained for *Os03g0244200*, with significant differences in both GPC and taste value among haplotypes (Figure 6d). Hap7 had the lowest GPC (7.78%), whereas Hap5 exhibited the highest taste value (81.77). Hap10 also showed a relatively low GPC (7.82%), although it was represented by comparatively few accessions. Unlike the other three candidate genes, favorable values for GPC and eating quality were not concentrated within a single major haplotype of *Os03g0244200*.

### 2.5. Haplotype Analysis of Known Genes

**Figure 7:**
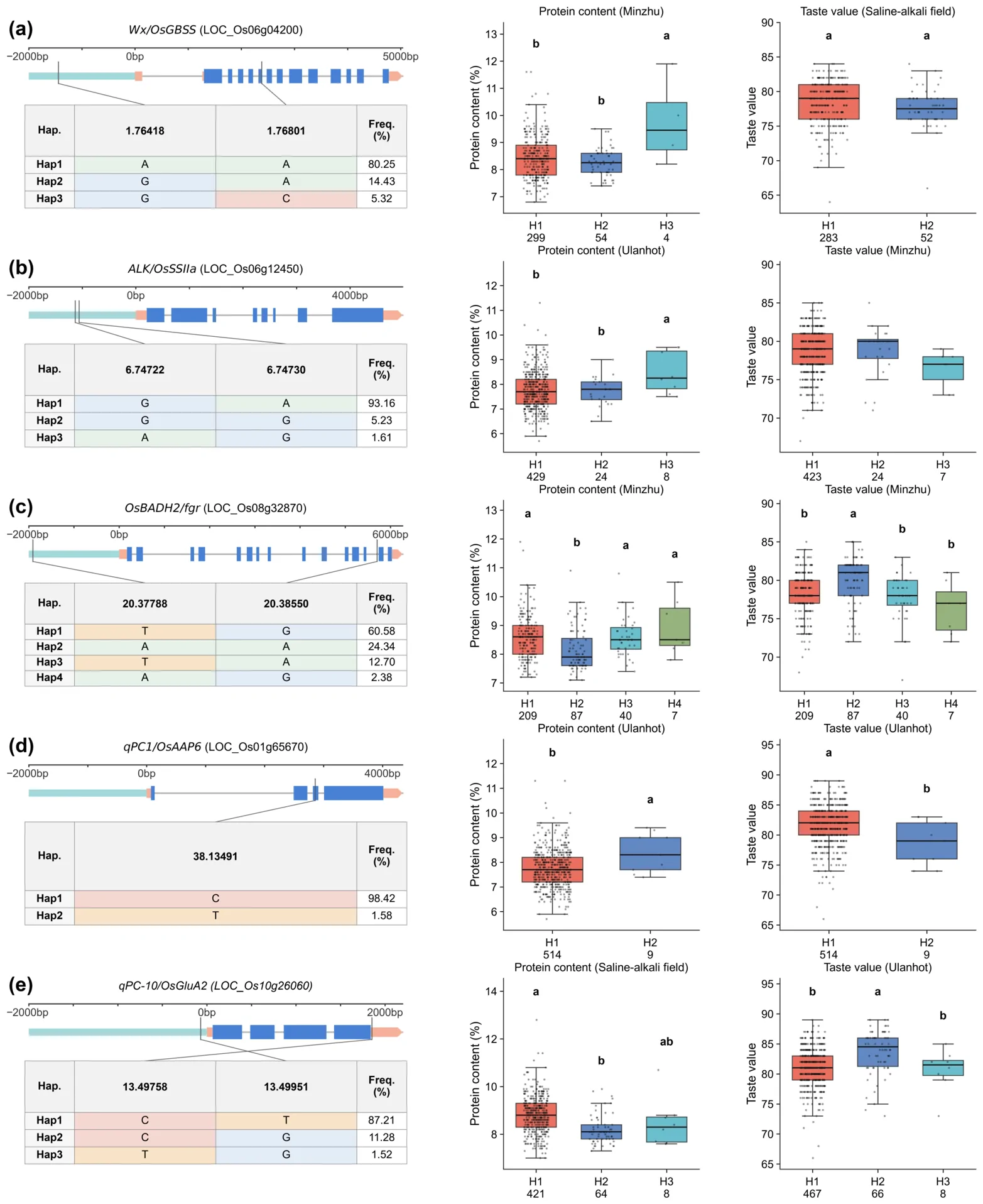
Haplotype effects of previously reported quality- and GPC-related genes. (**a**–**e**) *Wx/OsGBSS*, *ALK/OsSSIIa*, *OsBADH2/fgr*, *qPC1/OsAAP6*, and *qPC-10/OsGluA2*, respectively. Selected comparisons are shown, with the site labeled above each boxplot; complete results for all three sites are provided in Figures S2–S4. Different lowercase letters indicate significant differences at *p* < 0.05. Colored cells in the haplotype tables distinguish allele patterns. Box colors distinguish haplotype groups within each panel, and dots represent individual observations.

To place the newly identified candidate loci in the context of established rice quality genes, haplotype variation was further examined for five previously reported quality- or GPC-related genes, including *Wx/OsGBSS, ALK/OsSSIIa, OsBADH2/fgr, qPC1/OsAAP6, and qPC-10/OsGluA2*. The phenotypic effects of these haplotypes on GPC and taste values varied among genes and experimental sites (Figure 7 and Figures S2–S4).

Three haplotypes were identified for *Wx/OsGBSS*. At Harbin Minzhu, GPC differed significantly among haplotypes (Figure 7a and Figure S2a), whereas no significant differences in taste value were observed (Figure S2a). No significant differences in GPC or taste value were observed among *Wx/OsGBSS* haplotypes at Ulanhot (Figure S4a). Across the three sites, haplotypes associated with lower GPC did not consistently correspond to higher taste values (Figures S2a–S4a).

Three haplotypes were identified for *ALK/OsSSIIa*. At Harbin Minzhu, GPC differed significantly among haplotypes, with values of 8.47%, 8.58%, and 9.20% for Hap1, Hap2, and Hap3, respectively (Figure S2b). Their corresponding taste values were 78.54, 78.62, and 76.43 (Figure 7b and Figure S2b). At Ulanhot, Hap3 showed significantly higher GPC than Hap1 and Hap2, whereas no significant difference in taste value was observed (Figure 7b and Figure S4b). No clear phenotypic differences among haplotypes were observed at the saline–alkali site (Figure S3b).

Four haplotypes were identified for *OsBADH2*/*fgr*, and significant differences in both traits were observed at Harbin Minzhu (Figure 7c). Among them, Hap2 had the lowest GPC (8.16%) and the highest taste value (79.99). Two haplotypes were identified for *qPC1*/*OsAAP6*. At Harbin Minzhu, GPC did not differ significantly between the two haplotypes, whereas the taste value was significantly higher for Hap1 than for Hap2 (Figure 7d). At Ulanhot, the two haplotypes differed significantly in both GPC and taste value (Figure S4), whereas no clear differences were observed at the saline–alkali site.

Three haplotypes were identified for *qPC-10/OsGluA2*. At Harbin Minzhu, significant differences were observed among haplotypes for both GPC and taste value. Hap1, Hap2, and Hap3 had GPC values of 8.54%, 8.19%, and 8.04%, respectively, with corresponding taste values of 78.36, 79.77, and 79.43. Hap2 showed significantly lower GPC and a higher taste value than Hap1, whereas Hap3 showed intermediate values in the multiple-comparison test (Figure 7e). Across the candidate and previously reported genes, a separate population- and kinship-adjusted sensitivity analysis identified six significant associations among 48 testable gene-trait-site combinations after Benjamini–Hochberg correction (q < 0.05). These included *Os11g0239500* at Harbin Minzhu (GPC and taste value), *Os07g0264100* at Ulanhot (GPC and taste value) and the saline–alkali site (taste value), and *Os03g0244200* at the saline–alkali site (taste value). Thus, selected associations remained supported after adjustment, whereas other unadjusted differences were sensitive to population-background correction (Table S2).

### 2.6. Joint Haplotype Classification of *Os11g0239500* and *Os07g0264100*

Based on the single-gene haplotype analyses, *Os11g0239500* and *Os07g0264100* were selected for joint haplotype classification because their haplotypes showed contrasting phenotypic patterns for GPC and eating quality. Hap2, Hap3, and Hap4 of *Os11g0239500* were associated with relatively low GPC and high taste values at Harbin Minzhu. For *Os07g0264100*, Hap1 showed significantly lower GPC and a higher taste value than Hap2, whereas Hap3 showed the same directional trend but belonged to an intermediate significance group. Hap1 and Hap3 were therefore grouped together for exploratory joint classification based on their similar phenotypic direction; this grouping was not intended to imply an independently significant favorable effect of Hap3. *Os11g0239900* was not included because relatively few accessions were available for the joint analysis, whereas *Os03g0244200* was excluded because lower GPC and higher eating quality were not concentrated within the same major haplotype.

**Figure 8:**
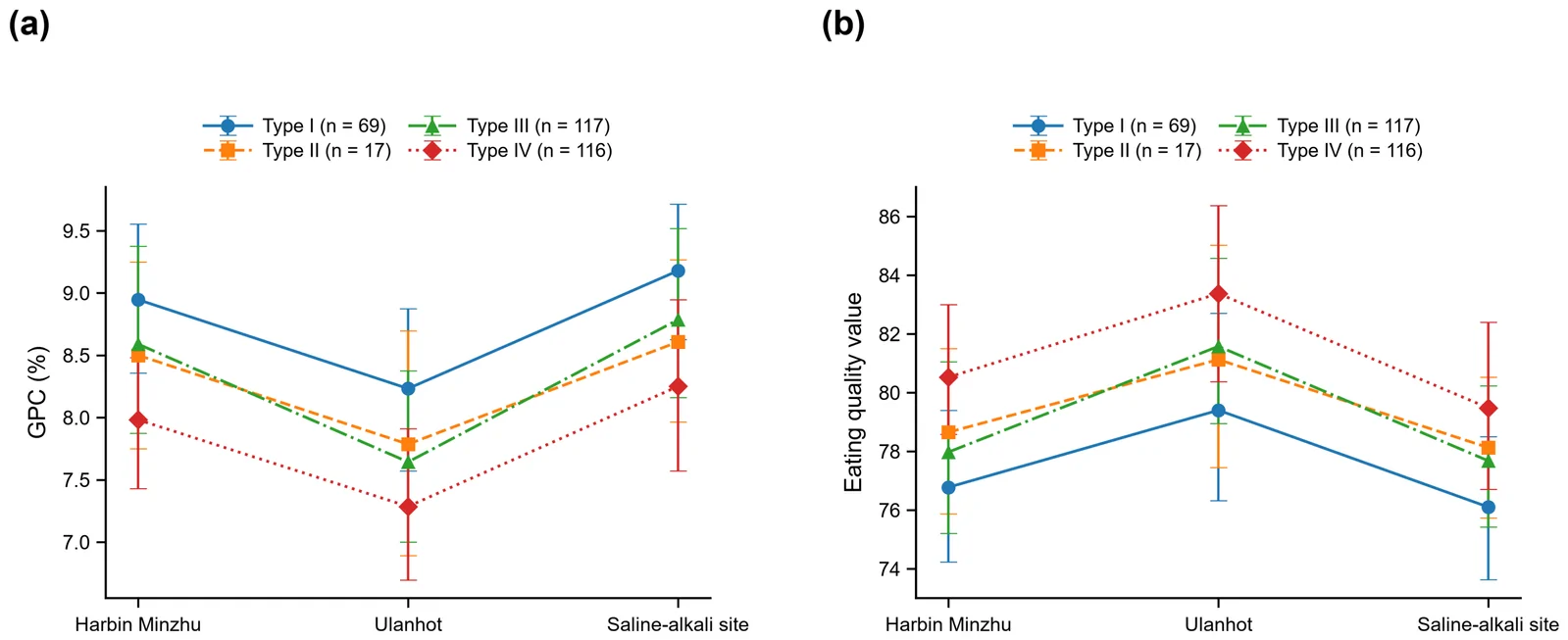
Phenotypic performance of four joint haplotype combinations of *Os11g0239500* and *Os07g0264100* across three experimental sites. (**a**) Grain protein content (GPC) of the four joint haplotype types at Harbin Minzhu, Ulanhot, and the saline–alkali site. (**b**) Taste values of the four joint haplotype types at the three experimental sites. Points represent mean values, and error bars indicate standard deviations (SD). Types I–IV correspond to the joint haplotype combinations defined in Table 3.

In the original descriptive analysis, after genotype and phenotype filtering (Section 4.6), 319 accessions were assigned to four joint haplotype types (Types I–IV: *n* = 69, 17, 117, and 116, respectively; Table 3). Their mean GPC values across sites were 8.78%, 8.30%, 8.38%, and 7.84%, respectively, and their mean taste values were 77.45, 79.35, 79.23, and 81.14. Differences among types were significant for both traits based on accession means averaged across the three sites (*p* < 0.001). The site-specific GPC and taste-value patterns for the four joint haplotype types are illustrated in Figure 8.

In site-specific comparisons, Type IV had lower GPC than Type I at Harbin Minzhu (mean difference, −0.97 percentage points; 95% CI, −1.15 to −0.80), Ulanhot (−0.93; 95% CI, −1.12 to −0.73), and the saline–alkali site (−0.93; 95% CI, −1.11 to −0.76). Type IV also had higher taste values at these sites, with mean differences of 3.74 points (95% CI, 2.98 to 4.51), 3.86 points (95% CI, 2.93 to 4.79), and 3.46 points (95% CI, 2.69 to 4.24), respectively. All six comparisons remained significant after Holm adjustment (adjusted *p* < 0.001). These findings indicate associations between joint haplotype type and the two traits but do not establish additive or interactive effects between *Os11g0239500* and *Os07g0264100*.

As a separate population- and kinship-adjusted sensitivity analysis, 189 accessions with complete validated genotypes and phenotypic observations were retained under stricter filtering criteria (Types I-IV: *n* = 37, 11, 61, and 80, respectively). At Harbin Minzhu, Type IV had lower adjusted GPC than Type I (difference = −0.691 percentage points; Holm-adjusted *p* = 0.023) and a higher adjusted taste value (difference = 2.747 points; Holm-adjusted *p* = 0.030). The corresponding predefined Type IV–Type I contrasts at Ulanhot and the saline–alkali site were not statistically significant after adjustment. These findings support the joint haplotype association at Harbin Minzhu, with less consistent evidence across sites (Table S2).

## 3. Discussion

### 3.1. Environmental Variation in GWAS Signals

Although GPC was significantly correlated among the three experimental sites, the major association signals showed distinct site-dependent patterns. At Harbin Minzhu, significant associations were concentrated primarily within the 6.92–7.50 Mb interval on chromosome 11, whereas most association signals detected at Ulanhot and the saline–alkali site were located on chromosome 7. GPC is a complex quantitative trait with a genetic basis [14], and environmental conditions can influence rice grain quality and protein composition [25,26]. The differences in GWAS signals among the three experimental sites may partly reflect environmental variation, although other factors may also contribute. The separate site-specific GWAS analyses did not formally test SNP × site interactions; thus, these patterns should not be interpreted as established locus-specific G×E effects.

Chromosome 7 has been repeatedly implicated in the genetic regulation of rice GPC. In the present population, a cluster of significant associations on chromosome 7 was detected at Ulanhot, and regional association and LD analyses highlighted *Os07g0264100* as a candidate gene. Chen et al. [14] reported *qPC7.2*, with the lead SNP sf0709202668 at chr7:9,202,668 bp on the MSU Nipponbare v6.1 assembly. Sequence-based coordinate conversion mapped this SNP to chr7:9,203,663 bp on the IRGSP-1.0 assembly, approximately 149.7 kb from the significant SNP identified within *Os07g0264100* at chr7:9,353,384 bp in the present study. These are nearby signals, but we do not consider them the same locus. Alam et al. [27] reported *qGPC7.2*.

A distinct association pattern was observed on chromosome 11. At Harbin Minzhu, the 6.92–7.50 Mb interval formed a prominent association region containing several candidate genes, including *Os11g0239000*, *Os11g0239200*, *Os11g0239400*, *Os11g0239500*, *Os11g0239900*, and *Os11g0240550*. Haplotype analyses further revealed phenotypic differences in GPC and eating quality for several genes within this interval. Because these genes are physically close and may be in linkage disequilibrium, the observed haplotype associations cannot yet be attributed to a single causal gene. Therefore, the chromosome 11 region should be regarded as a major candidate interval for GPC variation in cold-region japonica rice, with further functional studies required to distinguish the contributions of individual genes. The additional GEMMA analysis detected significant associations on chromosome 7 across all three experimental sites, whereas the prominent chromosome 11 signals identified by GLM-Q did not remain genome-wide significant. These results suggest that the chromosome 11 associations were more sensitive to model selection and should be interpreted with caution.

Nevertheless, the additional population- and kinship-adjusted haplotype analysis retained significant overall associations of *Os11g0239500* with GPC and instrument-derived taste value at Harbin Minzhu. The *Os11g0239500* haplotype-by-site interaction was significant for taste value after BH correction (*q* = 0.045), but not for GPC (*q* = 0.112). Thus, the taste-value association showed evidence of site dependence, whereas a locus-specific G × E effect on GPC was not established. Together with the GEMMA results, these observations support cautious prioritization of *Os11g0239500* for further investigation.

### 3.2. Haplotype Effects in Cold-Region Japonica Rice

The haplotype patterns of the GWAS candidate genes were broadly consistent with the site-specific distribution of the association signals. For the chromosome 11 candidate genes *Os11g0239500* and *Os11g0239900*, significant haplotype differences were detected mainly at Harbin Minzhu. In particular, Hap2, Hap3, and Hap4 of *Os11g0239500* were associated with relatively low GPC and high taste values. By contrast, haplotype differences for *Os07g0264100* were most evident at Ulanhot, where Hap1 differed significantly from Hap2 in the favorable direction for both traits, whereas Hap3 showed a similar numerical trend but an intermediate multiple comparison grouping. These observations are consistent with the site-specific association patterns observed for the chromosome 11 and chromosome 7 regions. After adjustment for population structure and kinship, *Os07g0264100* retained significant overall haplotype associations with both traits at Ulanhot and with taste value at the saline–alkali site. By contrast, the adjusted associations of *Os11g0239500* remained significant only at Harbin Minzhu. This contrast suggests greater robustness of selected chromosome 7 haplotype associations, without establishing causal effects for either candidate locus.

The effects of previously reported GPC-related genes were not fully conserved in the present cold-region japonica population. *OsAAP6* encodes an amino acid transporter involved in amino acid allocation and grain protein accumulation [9,10]. However, the *qPC1/OsAAP6* haplotypes showed no significant difference in GPC at Harbin Minzhu, while Hap1 had a significantly higher taste value than Hap2. This difference was not consistently detected at the other two sites. In contrast, natural variation in the promoter region of *OsGluA2* has previously been associated with differences in gene expression and GPC [12,13]. At Harbin Minzhu, Hap2 of *qPC-10/OsGluA2* showed significantly lower GPC and a higher taste value than Hap1, whereas Hap3 showed intermediate values in the multiple comparison test. Recent integrative transcriptogenomic analysis further supports a close connection between storage protein regulation and starch and sucrose related pathways underlying rice eating and cooking quality [28]. These contrasting patterns suggest that the phenotypic effects of known GPC related genes may depend on allele frequencies, linkage backgrounds, and the overall genetic composition of the target population.

A similar pattern was observed for established genes related to eating and cooking quality. *Wx*/*OsGBSS* and *ALK*/*OsSSIIa* are major determinants of amylose content and gelatinization properties, respectively [29,30], whereas *OsBADH2*/*fgr* is a key gene controlling rice fragrance [31]. Although some haplotypes of *Wx* and *ALK* differed in GPC, lower GPC was not consistently accompanied by higher taste values across the three experimental sites. Hap2 of *OsBADH2*/*fgr* showed both lower GPC and higher eating quality at Harbin Minzhu; however, because *OsBADH2* is primarily associated with fragrance, the observed GPC difference is more likely to reflect linked genetic variation or differences in haplotype background rather than a direct effect of *OsBADH2* on protein accumulation. Storage protein composition itself can also influence rice flavor and sensory properties [32].

Collectively, these results indicate that previously characterized gene functions alone may not be sufficient to predict the phenotypic performance of specific haplotypes in a breeding population. Long-term selection for adaptation, maturity, yield, and grain quality may have altered the frequencies and combinations of major quality related alleles in cold-region japonica rice. Therefore, population specific evaluation of haplotype effects may provide complementary information for identifying favorable genetic variation and assessing its potential value in quality-oriented breeding.

### 3.3. Phenotypic Variation Among Joint Haplotype Classes

The joint haplotype analysis revealed a clear phenotypic gradient among the four classes. Type IV showed the lowest mean GPC and the highest mean taste value, whereas Types II and III exhibited intermediate phenotypic values. However, these observations should not be interpreted as evidence for additive or epistatic effects between *Os11g0239500* and *Os07g0264100*. The present analysis was based on predefined joint haplotype classes, and the class sizes were unbalanced, particularly for Type II (*n* = 17). Therefore, the relative contributions of the two loci and their potential interaction cannot be distinguished from the current analysis. The additional population- and kinship-adjusted sensitivity analysis found that the predefined Type IV–Type I differences in GPC and taste value remained significant at Harbin Minzhu but not at the other two sites. These findings indicate that the original joint haplotype patterns are exploratory associations whose statistical support varies with both site and adjustment for population background.

The relationship between GPC and eating quality has previously been examined in several rice populations. For example, favorable natural variation at *Ghd7.1* has been reported to reduce GPC while improving eating quality [6], indicating that these two traits are not necessarily genetically inseparable. Recent association studies have likewise highlighted the value of considering GPC together with other grain-quality traits when identifying breeding targets [21,23,33]. Takeuchi et al [34] used two chromosome segment substitution lines in the Koshihikari background to confirm that Satojiman-derived segments carrying eating-quality QTLs on chromosomes 4 and 11 increased eating-quality scores. In the present study, the intermediate phenotypic values of Types II and III and the lower GPC and higher taste value observed for Type IV indicate an association between joint haplotype classification and phenotypic performance. However, the present analysis cannot determine whether this pattern reflects additive effects, locus-by-locus interaction, or the predominant contribution of one locus.

Importantly, the present results demonstrate an association rather than a direct causal relationship between these haplotypes and the observed phenotypes. *Os11g0239500* and *Os07g0264100* should therefore be regarded as candidate loci for further investigation rather than functionally validated regulators of GPC or eating quality. Nevertheless, the consistent phenotypic tendency of Type IV across the three experimental sites identifies this joint haplotype class as a potentially useful target for further validation in independent germplasm populations.

Several limitations should also be considered. The phenotypic data were obtained from three experimental sites within a single growing season in 2023, and the stability of the observed joint haplotype associations remains to be evaluated across additional years and independent germplasm populations. The broad-sense heritability estimate was obtained using a multi-environment mixed model that separately accounted for genotype × environment interaction variance. Further statistical evaluation and functional validation will therefore be required to clarify the genetic basis of the associations observed for *Os11g0239500*, *Os07g0264100*, and the chromosome 11 candidate interval.

## 4. Materials and Methods

### 4.1. Plant Materials and Field Experiments

A total of 624 cold-region japonica rice accessions were used in this study. The accessions were obtained from the germplasm repository of the Institute of Crop Cultivation and Tillage, Heilongjiang Academy of Agricultural Sciences, and consisted mainly of released cultivars, stable breeding lines, and other japonica rice germplasm (Table S1). Field experiments were conducted in 2023 at three sites in northeastern China: the Minzhu experimental site in Harbin, Heilongjiang Province (45°49’ N, 126°50’ E); a rice research field of the Hinggan League Institute of Agricultural and Animal Husbandry Sciences in Xibaiyin Gacha, Ulanhot, Inner Mongolia Autonomous Region (46.13178974° N, 122.09580715° E); and a saline–alkali experimental field in Baiyintala Gacha, Bayannur Sumu, Horqin Right Wing Middle Banner, Hinggan League, Inner Mongolia Autonomous Region (44.74908853° N, 121.89089326° E). Soil conditions at the saline–alkali experimental site were monitored at four stages: before land preparation, tillering, heading, and after harvest. The recorded soil pH ranged from 8.05 to 8.72, exchangeable sodium percentage (ESP) from 13.59% to 19.62%, total salt content from 1.43 to 2.97 g/kg, and soil organic matter content from 20.2 to 21.3 g/kg. These measurements indicated alkaline and saline soil conditions at this experimental site.

Seeds were sown on 15 April, and seedlings were transplanted on 23 May. One seedling was transplanted per hill, and each accession was planted in four rows, each 2 m in length. Each accession was evaluated in three independent field replicate plots at each experimental site. At the saline–alkali site, each replicate plot occupied 1.08 m^2^. The individual field plot was considered the experimental unit. Field management, including water and fertilizer management and the control of diseases, insect pests, and weeds, followed local rice production practices. All accessions within each site were managed under the same field conditions. At maturity, each plot was harvested separately, and the grains were naturally air-dried before GPC and eating quality measurements. Of the 624 accessions, 495 had complete GPC phenotypic data across all three sites and genotype data that passed quality control and were therefore used for GWAS.

### 4.2. Measurement of Grain Protein Content and Eating Quality

After natural air-drying, the grains were threshed, dehulled, and milled. Grain protein content (GPC) was measured using a JSWL-200 rice taste analyzer (Beijing Dongfu Jiuheng Instrument Technology Co., Ltd., Beijing, China) based on near-infrared spectroscopy. Each sample was measured three times, and the mean value was used as the GPC phenotype for the corresponding experimental site. These three measurements were technical replicates of the same prepared grain sample and were not considered independent biological replicates. Protein content was also determined in representative samples using the Kjeldahl method according to GB 5009.5-2016 [35], and the results were used to verify the reliability of the near-infrared measurements. Taste values were obtained using the preset program of the same instrument and were used as an instrumental index of eating quality. The available technical documentation does not clarify whether the protein content reported by the JSWL-200 is directly used as an input in its taste-value calculation. Therefore, potential algorithmic dependence between the two instrument-derived traits cannot be excluded, and their association should be interpreted cautiously. Samples from all three experimental sites were processed and measured under the same conditions.

### 4.3. Genome Resequencing and Variant Calling

Leaf samples were collected from the rice accessions described in Section 4.1. Genomic DNA was extracted using the cetyltrimethylammonium bromide method. DNA integrity was examined by agarose gel electrophoresis, and DNA concentration was measured using a Qubit 2.0 fluorometer. Sequencing libraries with insert sizes of 300–350 bp were prepared using the GenoBaits DNA Library Prep Kit for ILM. Paired-end sequencing was performed on the MGI-2000/MGI-T7 platform, generating 150 bp reads at an average depth of approximately 10×. Raw reads were filtered using fastp v0.20.0 with the parameters -n 10, -q 20, and -u 40 [36]. The filtered paired-end reads were aligned to the *Oryza sativa* cv. Nipponbare reference genome (IRGSP-1.0) using BWA-MEM (BWA v0.7.17-r1188) with default parameters, including a minimum seed length of 19 bp, a match score of 1, a mismatch penalty of 4, a gap-opening penalty of 6, and a gap-extension penalty of 1 [37]. SNPs and InDels were called using GATK v4.0.4.0 HaplotypeCaller [38]. Hard filtering was performed using VariantFiltration with the following thresholds: QD < 2.0, QUAL < 30.0, MQ < 40.0, FS > 60.0, SOR > 3.0, MQRankSum < −12.5, and ReadPosRankSum < −8.0. Variants were annotated using ANNOVAR (version 2020Jun08) [39]. Of the 5,518,860 SNPs initially identified, 1,527,603 were retained after quality control using PLINK [40], excluding SNPs with a minor allele frequency ≤ 0.05 or genotype missingness ≥ 10%.

### 4.4. Population Structure and Linkage Disequilibrium Analysis

Population structure was analyzed using ADMIXTURE [41], with K values ranging from 1 to 15. The cross-validation error progressively decreased across the tested K values and reached its minimum at K = 15. Therefore, the Q matrix obtained at K = 15 was used as the population-structure covariate in the subsequent association analysis (Figure S1). Accessions with an individual genotype missing rate greater than 10% were excluded from PCA, leaving 425 accessions for analysis. K means clustering was performed based on PC1-PC3 with k = 2. A kinship matrix was calculated using genome-wide SNPs to evaluate genetic relationships among the accessions. Linkage disequilibrium decay was estimated using PopLDdecay [42], and the physical distance at which the mean *r*^2^ decreased to half of its initial value was defined as the LD decay distance.

### 4.5. GWAS and Candidate Gene Identification

Association analysis was performed using TASSEL v5.2 with the GLM-Q model, including the population-structure Q matrix obtained from ADMIXTURE at K = 15 as a covariate. To further evaluate the robustness of the GWAS results, an additional association analysis was conducted using the linear mixed model implemented in GEMMA to account for genetic relatedness among accessions. Genomic inflation factors (λGC) were calculated from genome-wide association *p* values for each model at each experimental site to assess potential inflation of the association test statistics. The same genome-wide significance threshold of $-\log_{10}\left(P\right)>7.5$, corresponding to *p* < $3.16\times 10^{-8}$, was applied to both models. This cutoff is slightly more stringent than the Bonferroni-corrected threshold based on 1,527,603 tested SNPs. Manhattan and Q-Q plots were generated to visualize and compare the GWAS results.

Significant SNPs were annotated individually according to their genomic positions relative to annotated rice genes and were not grouped into LD-defined loci. Gene-body variants were classified according to their predicted genomic consequences. Upstream and downstream variants were defined as those located within 2 kb of the transcription start and end sites, respectively, accounting for gene orientation. The estimated genome-wide LD decay distance of approximately 300 kb was used to characterize population LD and was not applied as a fixed window for SNP grouping or candidate-gene identification. Candidate genes were compiled from the annotation results of significant GWAS SNPs, including gene assignments associated with intergenic variants. After removing duplicate gene IDs, 28 nonredundant positional candidate genes were identified.

Four representative genes were selected based on association strength, genomic distribution, and variant annotation: *Os11g0239500* and *Os11g0239900* from the major chromosome 11 association region, and *Os07g0264100* and *Os03g0244200*, each containing a significant intronic SNP on chromosomes 7 and 3, respectively. Regional LD analyses were subsequently performed to examine local linkage patterns around these candidates (Figure 5).

### 4.6. Haplotype Analysis

Haplotype analysis was performed using SNP data from whole-genome resequencing of the 624 cold-region japonica rice accessions. Polymorphic SNPs within *Os11g0239500*, *Os11g0239900*, *Os07g0264100*, and *Os03g0244200* and their flanking regions were used to define haplotypes based on allele combinations. Haplotype patterns differing only at missing genotype positions were combined when their observed alleles were consistent. For individual-gene haplotype analyses, groups containing fewer than four accessions were excluded from subsequent phenotypic comparisons. GPC and taste values were compared among the retained haplotypes separately at each experimental site. Haplotype analyses were also conducted for five previously reported quality- or GPC-related genes, including *Wx/OsGBSS, ALK/OsSSIIa, OsBADH2/fgr, qPC1/OsAAP6*, and *qPC-10/OsGluA2*. Joint haplotype analysis was performed for *Os11g0239500* and *Os07g0264100*. Of the 624 accessions, 216 lacked valid genotype calls at one or both loci, leaving 408. Among these, 75 were excluded because at least one phenotypic value was missing across the three sites, and a further 14 were excluded because zero-coded phenotypic values were treated as missing. The remaining 319 accessions were assigned to four joint haplotype types (Types I–IV: *n* = 69, 17, 117, and 116, respectively). No additional accessions were excluded during grouping. GPC and taste values were then compared among the four types.

For the separate population-adjusted sensitivity analysis, we required complete VCF-validated genotype calls and retained haplotype groups only if they contained at least 10 accessions and represented at least 5% of the callable accessions for the corresponding gene. These criteria were stricter than those used for the original descriptive analysis. After genotype and phenotype filtering, 189 accessions were retained for the adjusted four-type joint haplotype sensitivity analysis (Types I–IV: *n* = 37, 11, 61, and 80, respectively).

### 4.7. Statistical Analysis

The mean, standard deviation (SD), range, and coefficient of variation (CV) of GPC were calculated for each of the three experimental sites. The correlation of GPC across sites was evaluated using Pearson correlation coefficients. The entry-mean broad-sense heritability (*H*^2^) of GPC across the three experimental sites was re-estimated using a multi-environment linear mixed-effects model. Site was treated as a fixed effect, whereas genotype and genotype × site interaction were fitted as random effects. Variance components were estimated by restricted maximum likelihood (REML). The analysis was based on independently replicated field-plot observations rather than repeated instrumental measurements. Entry-mean broad-sense heritability was calculated using the following formula:

(1) $$H^{2}=\frac{\sigma_{G}^{2}}{\sigma_{G}^{2}+\frac{\sigma_{G\times E}^{2}}{n_{E}}+\frac{\sigma_{e}^{2}}{n_{E}r}}$$

where $\sigma_{G}^{2}$ represents the genotypic variance, $\sigma_{G\times E}^{2}$ represents the genotype × environment interaction variance, and $\sigma_{e}^{2}$ represents the residual variance. The number of environments ($n_{E}$) and independent field replicates per environment (*r*) were both three. The estimated variance components were 0.313998, 0.472811, and 0.015834, respectively, resulting in an entry-mean broad-sense heritability of 0.663. For individual-gene haplotype analyses, differences in GPC and taste value were tested separately at each site using one-way ANOVA, including haplotypes represented by at least four accessions. For the original descriptive analyses, nominally significant omnibus tests (*p* < 0.05) were followed by Tukey’s HSD test. Overall differences among joint haplotype types were tested by one-way ANOVA followed by Tukey’s HSD test, using accession means averaged across the three sites. For the original haplotype analyses, a total of 54 individual-gene omnibus tests and two joint-haplotype omnibus tests were conducted. The *p*-values from these 56 omnibus tests were adjusted using the Benjamini–Hochberg procedure to account for multiple testing. In addition, Type I and Type IV were compared separately at each site using Welch’s two-sample *t*-tests. Mean differences and 95% confidence intervals are reported, and *p* values across the six comparisons (three sites × two traits) were adjusted using the Holm method.

Additional haplotype association analyses were performed using linear mixed models incorporating the first five genetic principal components (PC1–PC5) as fixed covariates and a VanRaden genomic kinship matrix to account for genetic relatedness. The principal component covariates were derived from a separately quality-controlled genotype dataset. The omnibus *p*-values from 48 testable gene-trait-site associations were adjusted using the Benjamini–Hochberg procedure. Haplotype-by-site interactions were also evaluated in the population- and kinship-adjusted framework, with Benjamini–Hochberg correction applied to the 16 evaluable interaction tests. For the adjusted joint haplotype analysis, the predefined Type IV–Type I contrasts were evaluated separately at each site, and *p*-values were adjusted using the Holm procedure across the six site-by-trait comparisons (Table S2).

## 5. Conclusions

This study characterized the genetic variation underlying grain protein content (GPC) in a panel of cold-region japonica rice accessions evaluated across three experimental sites. Genome-wide association analysis identified major GPC-associated regions on chromosomes 11 and 7, and haplotype analyses highlighted *Os11g0239500*, *Os11g0239900, Os07g0264100,* and *Os03g0244200* as candidate loci for further investigation. Joint haplotype analysis of *Os11g0239500* and *Os07g0264100* indicated lower GPC and higher taste values for Type IV than Type I, with significant adjusted differences observed at Harbin Minzhu. These results identify GPC-associated regions and haplotype patterns for further investigation. Functional validation and evaluation in independent populations are needed to determine their potential value for rice breeding.

## Figures and Tables

**Table 1:** Summary statistics for grain protein content across three experimental sites.

Trait	Location	Mean (%)	SD	Median (%)	Min (%)	Max (%)	CV (%)	Skewness	Kurtosis
Protein	Minzhu	8.48	0.73	8.40	6.80	11.90	8.62	0.77	1.71
Protein	Saline–alkali site	8.70	0.76	8.70	7.00	12.80	8.70	0.57	1.45
Protein	Ulanhot	7.73	0.75	7.60	5.70	10.40	9.70	0.41	0.20

**Table 2:** Positional candidate genes identified by annotation of significant SNPs from the GLM-Q analysis.

Env.	Gene ID	Chr.	Position (bp)	*p*-Value	Alleles	Region	No. of SNPs
Minzhu	*Os11g0239500*	11	7,440,320	$2.61\times 10^{-10}$	A/G	Upstream	7
Minzhu	*Os11g0240550*	11	7,491,433	$4.56\times 10^{-10}$	T/C	NcRNA Exonic	3
Minzhu	*Os11g0239900*	11	7,472,700	$6.63\times 10^{-10}$	A/G	Intergenic	13
Minzhu	*Os11g0240600*	11	7,496,597	$3.88\times 10^{-9}$	A/T	Upstream	2
Minzhu	*Os11g0239200*	11	7,430,286	$7.36\times 10^{-9}$	T/C	Upstream	1
Minzhu	*Os11g0229700*	11	6,915,934	$8.43\times 10^{-9}$	A/G	Intergenic	1
Minzhu	*Os11g0239400*	11	7,435,598	$1.41\times 10^{-8}$	A/G	Upstream	2
Minzhu	*Os11g0239000*	11	7,425,877	$1.56\times 10^{-8}$	G/A	Intergenic	1
Minzhu	*Os03g0244200*	3	7,628,208	$2.71\times 10^{-8}$	T/C	Intronic	1
Ulanhot	*Os07g0252400*	7	8,504,071	$2.81\times 10^{-9}$	C/T	Intergenic	1
Ulanhot	*Os07g0270800*	7	9,967,834	$3.64\times 10^{-9}$	G/A	Intergenic	1
Ulanhot	*Os07g0260400*	7	9,115,104	$4.14\times 10^{-9}$	A/G	Intergenic	2
Ulanhot	*Os07g0265600*	7	9,484,064	$9.73\times 10^{-9}$	G/C	Intergenic	1
Ulanhot	*Os07g0284950*	7	10,951,257	$1.19\times 10^{-8}$	C/T	Intergenic	1
Ulanhot	*Os07g0277500*	7	10,434,403	$1.80\times 10^{-8}$	G/A	Intergenic	1
Ulanhot	*Os07g0419800*	7	13,437,093	$3.12\times 10^{-8}$	G/A	Exonic	2
Ulanhot	*Os07g0280200*	7	10,669,626	$1.85\times 10^{-8}$	G/A	Intergenic	1
Ulanhot	*Os10g0142100*	10	2,571,075	$1.92\times 10^{-8}$	G/A	Intronic	1
Ulanhot	*Os07g0414900*	7	13,096,099	$2.05\times 10^{-8}$	G/A	Intergenic	2
Ulanhot	*Os07g0267400*	7	9,578,789	$2.06\times 10^{-8}$	G/A	Intergenic	1
Ulanhot	*Os07g0264100*	7	9,353,384	$2.21\times 10^{-8}$	T/A	Intronic	1
Ulanhot	*Os06g0698674*	6	29,235,264	$2.22\times 10^{-8}$	T/C	Exonic	1
Ulanhot	*Os07g0256700*	7	8,832,176	$2.43\times 10^{-8}$	T/A	Downstream	2
Ulanhot	*Os07g0289800*	7	11,238,699	$2.43\times 10^{-8}$	C/A	Intergenic	1
saline–alkali	*Os07g0249200*	7	8,296,999	$5.28\times 10^{-9}$	G/A	Intergenic	1
saline–alkali	*Os07g0255300*	7	8,769,631	$5.78\times 10^{-9}$	A/T	Intergenic	3
saline–alkali	*Os07g0404300*	7	12,379,765	$1.52\times 10^{-8}$	G/C	Intergenic	1
saline–alkali	*Os07g0256300*	7	8,819,901	$1.55\times 10^{-8}$	C/T	Intergenic	1

**Table 3:** Joint haplotype analysis of *Os11g0239500* and *Os07g0264100*.

Type	*Os11g0239500*	*Os07g0264100*	N	GPC (%)	Taste Value
I	Hap1/Hap5/Hap6/Hap7/Hap8	Hap2	69	8.78 ± 0.44	77.45 ± 2.02
II	Hap2/Hap3/Hap4	Hap2	17	8.30 ± 0.69	79.35 ± 2.49
III	Hap1/Hap5/Hap6/Hap7/Hap8	Hap1/Hap3	117	8.38 ± 0.58	79.23 ± 2.11
IV	Hap2/Hap3/Hap4	Hap1/Hap3	116	7.84 ± 0.52	81.14 ± 2.25

Note: Values are means ± SD of accession means across the three experimental sites.

## Data Availability

The raw data supporting the conclusions of this study are available from the corresponding author upon reasonable request.

## Supplementary Materials

The following supporting information can be downloaded at: https://www.mdpi.com/article/10.3390/plants15213355/s1, Table S1: Origin and distribution of the 624 rice accessions; Table S2: Population- and kinship-adjusted haplotype sensitivity analyses. Figure S1: ADMIXTURE analysis of population structure: (a) cross-validation error for K values from 1 to 15 and (b) inferred ancestry proportions at K = 15. Each vertical bar represents an accession; colors indicate inferred ancestry components; Figure S2: Haplotype analyses of five previously reported quality- and GPC-related genes at Harbin Minzhu; Figure S3: Haplotype analyses of five previously reported quality- and GPC-related genes at the saline–alkali site; Figure S4: Haplotype analyses of five previously reported quality- and GPC-related genes at Ulanhot; Figure S5: Haplotype analyses of four candidate genes at Harbin Minzhu; Figure S6: Haplotype analyses of four candidate genes at the saline–alkali site; Figure S7: Haplotype analyses of four candidate genes at Ulanhot.
